# Influence of depression and interpersonal support on adherence to antiretroviral therapy among people living with HIV

**DOI:** 10.1186/s12981-023-00538-8

**Published:** 2023-06-29

**Authors:** Jerry John Nutor, Akua O. Gyamerah, Robert Kaba Alhassan, Henry Ofori Duah, Rachel G.A. Thompson, Natalie Wilson, Orlando Harris, Jose Gutierrez, Thomas J. Hoffmann, Monica Getahun, Glenn-Milo Santos

**Affiliations:** 1 Department of Family Health Care Nursing, School of Nursing, University of California San Francisco, San Francisco, CA USA; 2 Department of Community Health and Health Behavior, University of Buffalo, Buffalo, NY USA; 3 Centre for Health Policy and Implementation Research, Institute of Health Research, University of Health and Allied Sciences, Ho, Ghana; 4 College of Nursing, University of Cincinnati, Cincinnati, OH USA; 5 Language Center, College of Humanities, University of Ghana, Accra, Ghana; 6 Africa Interdisciplinary Research Institute, Accra, Ghana; 7 Department of Community Health Systems, School of Nursing, University of California San Francisco, San Francisco, CA USA; 8 Department of Epidemiology and Biostatistics, Office of Research, School of Nursing, University of California, San Francisco, San Francisco, CA USA; 9 Institute for Global Health Sciences, University of California, San Francisco, San Francisco, CA USA

**Keywords:** Interpersonal support, Adherence, Depression, Antiretroviral therapy

## Abstract

**Background:**

Poor adherence and under-utilization of antiretroviral therapy (ART) services have been major setbacks to achieving 95-95-95 policy goals in Sub-Saharan Africa. Social support and mental health challenges may serve as barriers to accessing and adhering to ART but are under-studied in low-income countries. The purpose of this study was to examine the association of interpersonal support and depression scores with adherence to ART among persons living with HIV (PLWH) in the Volta region of Ghana.

**Methods:**

We conducted a cross-sectional survey among 181 PLWH 18 years or older who receive care at an ART clinic between November 2021 and March 2022. The questionnaire included a 6-item simplified ART adherence scale, the 20-item Center for Epidemiologic Studies Depression Scale (CES-D), and the 12-item Interpersonal Support Evaluation List-12 (ISEL-12). We first used a chi-squared or Fisher’s exact test to assess the association between these and additional demographic variables with ART adherence status. We then built a stepwise multivariable logistic regression model to explain ART adherence.

**Results:**

ART adherence was 34%. The threshold for depression was met by 23% of participants, but it was not significantly associated with adherence in multivariate analysis(p = 0.25). High social support was reported by 48.1%, and associated with adherence (p = 0.033, aOR = 3.45, 95% CI = 1.09–5.88). Other factors associated with adherence included in the multivariable model included not disclosing HIV status (p = 0.044, aOR = 2.17, 95% CI = 1.03–4.54) and not living in an urban area (p = 0.00037, aOR = 0.24, 95% CI = 0.11–0.52).

**Conclusion:**

Interpersonal support, rural residence, and not disclosing HIV status were independent predictors of adherence to ART in the study area.

## Introduction

Sub-Saharan Africa (SSA) is disproportionately impacted by the global HIV pandemic, with nearly 1 in every 25 adults infected, accounting for more than two-thirds of the global population of people living with HIV (PLWH) [1, 2]. To improve the uptake of antiretroviral therapy (ART) and prevent new infections, in 2014, the UNAIDS organization set ambitious targets of “90-90-90” by 2020 and “95-95-95” by 2030 to accelerate efforts toward ending the AIDS pandemic by 2030 [3]. Initiatives related to these goals helped improved HIV outcomes: over half (54%) of PLWH in SSA were on ART by the end of 2016 compared to 35% in 2014 [1]. However, poor adherence and under-utilized ART services have been major setbacks to achieving these policy goals in SSA [4–8]. Factors underpinning poor adherence and service utilization are poorly understood in SSA.

In Ghana in particular, HIV stakeholders implemented the WHO roadmap, “*90-90-90: Treatment for All*” policy in 2016 with the goal of diagnosing 90% of all people living with HIV, placing 90% of diagnosed people on ART, and achieving 90% viral suppression in treated people by 2020 [9]. Similarly, the “*Test All*” policy was initiated to improve universal access to quality HIV testing and treatment. These initiatives contributed to the increase in HIV testing, from 1 million in 2016 to 1.2 million in 2017. However, significant gaps remain with only 20% of HIV positive adults on ART [9]. To close this gap, more research is needed to better understand the barriers to optimizing outcomes along the HIV care continuum.

The most effective way to reduce new infections and promote health for PLWH is administration of ART and adherence to ART. However, multiple factors can impede the HIV care continuum. In a recent systematic review of research in low-to-middle income countries, individual and health system level factors with high potential to influence ART adherence and service utilization, such as family structure, the burdensome ART regimens, route of administration, and attitudes about medication, and health care and environmental factors, such as rural versus urban location and missed clinic appointments were identified [10]. In addition, ART services such as routine monitoring of viral load and CD4 count, nutritional counseling, and general health assessments are important for health and wellbeing [11]. The most recent WHO guidelines recommend that initiation of effective, combination ART should not be delayed for people newly diagnosed with HIV [12].

Additionally, PLWH should remain in care for a lifetime, regardless of CD4 count or viral load [13, 14]. Many PLWH in low-resourced countries do not take full advantage of ART services because of individual factors (e.g., depression, internalized stigma, poor attitudes and beliefs, forgetting to take ART, ART side effects, financial difficulty) [7, 15−18], community factors (e.g., family and community norms or lack of social support) [19–21], and healthcare factors (e.g. inaccessibility of services) [22, 23]. Of these many factors, individual and community factors are the least studied but most amenable to modification through interventions [22]. Depression and social support have been reported in previous studies to influence adherence to ART among PLWH [24, 25]. For example, in a systematic rereview conducted by Gonzalez and colleagues, they found that depression was significantly associated with lower ART adherence, and remain consistent over time, among those reporting depression [26]. However, high social support networks is associated with retention in HIV care and ART adherence among PLWH [27]. Despite these mounting evidence in other SSA countries, little or none is known in Ghana about the influence of depression and social support on HIV treatment outcomes among PLWH in Ghana. Therefore, our study sought to examine the influence of interpersonal support and depression on adherence to ART among PLWH in the Volta region of Ghana.

## Methods

### Study setting and design

We conducted a cross-sectional survey of PLWH who were receiving ART for at least 6 months prior to the data collection. The study was conducted in the ART clinic at the Ho Teaching Hospital in the Volta Region of Ghana. The Volta region is one of the 16 administrative regions of Ghana with a population of over two (2) million people. It is bordered by Eastern, Greater Accra and Oti regions to the west, south and north respectively. It is also bordered by a neighboring country, Togo, to the east.

The Ho Teaching Hospital is the only teaching hospital in the Volta region of Ghana. The hospital is a 300-bed capacity hospital, 25 physicians and approximately100 nurses, averaging about 500 daily outpatient cases. The hospital is the main ART referral clinic in the Volta Region serving over 1,000 PLWH.

### Sampling and recruitment

Using purposive sampling, we recruited 181 PLWH who received care at the clinic between November 2021 and March 2022. PLWH were contacted in person by a nurse when they arrived for clinic visit to obtain their antiretroviral medication or for their regular appointment with their healthcare providers. The attending nurse discussed the study with potential participants. Those PLWH who agreed to participate in the study were contacted by a trained research assistant. The research assistant explained the purpose, benefits and risks, and confidentiality to potential participants. A copy of the study’s information sheet was given to the potential participants who could read. For the PLWH who could not read, study personnel explained the information sheet in a local language that they could understand. Those who agreed to participate in the study were assisted to sign a consent form. PLWH were eligible to participate if there were age 18 years or older, enrolled in ART for at least 6 months, and identified by clinic staff as either a high or low compliance to the services (defined by more or less than 50% clinic attendance in the last 6 months) or low (defined by 50% or less clinic attendance in the last 6 months). We excluded PLWH who were seriously ill, defined as hospitalization in the past one month.

### Data collection

Following consent, questionnaires were self-administered. If participants could not read or write, questions were read in the language they understood best, and responses recorded on the questionnaire by the research assistants. Questionnaires were administered using programmed tablets. Participants received Ghana Cedis (GHS) equivalence of $10 compensation after interview for their time and travel cost. Our response rate was 100%.

### Sample size determination

Since we were primarily interested in the impact of depression and social support on ART adherence, we assumed α = 0.025, a Bonferroni correction for two tests. Using Stata/IC v16.1 [28]. Assuming an ART adherence prevalence of 30%, and a dichotomized depression/social support score prevalence of 50%, with 160 people, the minimum detectable odds ratio is 3.1, equivalent to an approximately medium effect size [29]. We note that in a Brazil population, which has different rates of depression (32.5%) and much different adherence (86% of depressed showed adherence, 95% non-depressed showed adherence), had an odds ratio of 3.81 (95% CI = 1.32–11.02) for depressed vs. non-depressed being adherent [30].

### Measures

#### Dependent variables

The simplified medication adherence questionnaire [31] comprising of 6 different question items, was used to measure adherence: Item 1: *Do you always take your medication at the appropriate time?* (0 = No or 1 = Yes); Item 2: *When you feel bad, have you ever discontinued taking your medication?* (0 = No or 1 = Yes); Item 3: *Have you ever forgotten to take your medication?* (0 = No or 1 = Yes); Item 4; *Have you ever forgotten to take your medication during the weekend?* (0 = No or 1 = Yes); Item 5: *In the last week, how many times did you fail to take your antiretroviral drug?* (1 = Never,2 = 1–2 times,3 = 3–5 times, 4 = 6–10 times,5 = More than 10 times; Item 6: *Since your last visit, how many whole days have gone by in which you did not take your medication*? (Open ended). Item six was subsequently categorized as ≤ 2days or > 2days. A negative response to item 1, *or* a positive response to either item 2, 3, or 4, *or* > 2 doses missed in the past week, *or* > 2days of non-medication in the past week was considered as not adherent. The inter-item reliability test was performed, and the Cronbach’s alpha value was 0.61.

#### Independent variables

The main independent variables of interest were depression and interpersonal support. Depression was measured with the Center for Epidemiologic Studies Depression Scale (CES-D) [32]. The CES-D is a 20-item Likert type questionnaire with response options for each item ranging from 0 to 3. The cumulative score has a range of 0 to 60. High scores on the CES-D implies greater symptoms of depression. Total depression scores were also categorized with the cut-off point of 16 [33], with scores below this cut off point described as low risk and scores ≥ 16 described as high risk for depression. The inter-item reliability test was performed with an acceptable Cronbach’s alpha value of 0.87.

Interpersonal support was measured with the Interpersonal Support Evaluation List-12 (ISEL-12) [34]. The ISEL-12 is a 12-item, 4-point Likert tool that measures perceived social support by asking respondents if they will be able to find assistance in 12 different social conditions of need. The responses are 1 = *definitely false*, 2 = *probably false*, 3 = *probably true*, and 4 = *definitely true.* Six items were reverse scored (Items 1, 2, 7, 8, 11, and 12). The ISEL-12 is further divided into three subscales including *Appraisal Support* (Items 2, 4, 6, and 11), *Belonging Support* (Items 1, 5, 7, and 9), and *Tangible Support* (Items 3, 8, 10, and 12). A higher score under each domain indicates higher levels of perceived social support. The median total score in this sample was 17, and scores below the median were categorized as low interpersonal support and scores ≥ 17 as high interpersonal support. The inter-item reliability test was performed for the total ISEL-12 scale and the sub-scales. Cronbach’s alpha values were 0.82-total scale, 0.63- *Appraisal Support*, 0.70- *Belonging Support*, and 0.52- *Tangible Support*.

Both the CES-D and the ISEL-12 measures were standardized (the mean was subtracted, and then that value was divided by the standard deviation) prior to performing multiple logistic regression.

#### Control variables

The following variables were considered as potential covariates: relationship status, place of residence, gender, age, level of education, monthly income, and disclosure of HIV status to partner (details of the levels for the variables can be found in Table 1).

**Table 1:** Characteristics of people living with HIV/AIDS in the Volta region of Ghana, 2022

Variables	n	%
Gender		
Female	24	15.1
Male	95	59.7
Missing	40	25.2
Age (years)		
<30	13	8.2
30–39	34	21.4
40–49	48	30.2
50–59	42	26.4
60+	22	13.8
Relationship Status		
Single/Widowed	71	44.7
In Relationship	87	54.7
Missing	1	0.6
Residence		
Rural	77	48.4
Urban	82	51.6
Level of formal education		
None	17	10.7
Primary	76	47.8
Secondary	45	28.3
Post-Secondary	20	12.6
Missing	1	0.6
Monthly Income		
≤1000GH (ref)	123	77.4
>1000GH	21	13.2
Missing	15	9.4
HIV disclosure to partner		
Disclosed	74	46.5
Have not disclosed	81	50.9
Missing	4	2.5
Total Depression Score		
Not-depressed (< 16)	123	77.4
Depressed (≥ 16)	36	22.6
Interpersonal support (ISEL-12)		
Low (< 17)	76	47.8
High (≥ 17)	83	52.2

### Statistical analysis

First, descriptive statistics were performed on each of the variables of interest using frequency tables (frequency and percentages). Second, we tested for association between each independent variable and adherence using a chi-square (or Fisher’s exact test when the cell sizes were small). Missing values were excluded from the chi-square test, but a separate Fisher’s exact test was run to determine if the proportion of missing was different among adherent and non-adherent groups. All aforementioned analyses was performed using Stata/IC version 14 [35]. Statistical significance for our primary analysis of depression and social support on adherence was set at p < 0.025 (Bonferroni correction for two tests), and for looking at other control covariates was set at p < 0.0056 (Bonferroni correction for 9 tests). We also noted tests with a nominal p < 0.05.

Third, to control for potential confounders, we then fit a stepwise multiple logistic regression model via the AIC criterion [36], after first forcing in our two independent variables of most interest (depression and interpersonal support). To utilize all individuals with non-missing adherence outcomes, we utilized a multiple imputation by chained equations (MICE) approach (with 100 imputations) using the package mice v3.13.0 [37] in R v4.1.0 [38]. In detail, MICE is a well-established flexible method for appropriately handling missing data, where each incomplete variable is imputed by a separate model; the process is run multiple times (i.e., 100 imputations here), with each imputation analyzed, and the results pooled [39]. We used polytomous (for categorical) and logistic (for binary) regression models for imputation [37]. Work has suggested that even larger amounts of missing data can be better modeled via multiple imputation; [40] we also note that variables with > 10% missing data were not brought into the final model. Finally, to assess how well the imputation worked, we looked at the imputation distribution of these covariates, which were similar to that of the data itself (e.g., male gender had observed prevalence 20.1%, and the IQR in the imputed dataset was 19.5-20.5%; income > 1000GH had observed prevalence 14.6%, and the IQR of the prevalence in the imputed datasets was 13.8-15.1%).

## Results

### Characteristics of study population

A total of 181 respondents filled out the survey however, 22 (12.2%) participated had their ART adherence data missing and were excluded from further analysis. Among the 159 PLWH included in the study, there were a significant proportion with missing data on independent variables (Table 1). Considering the available data, 79.8% were males, with an average age of the of 46.3 years (± 12.2 standard deviation). About half resided in urban areas, and the same proportion of participants were in a relationship. The majority (85.4%) had a monthly income of below GHC 1,000 (equivalent to USD 110). A total of 58.9% had studied up to elementary school. Only 47.7% of the respondents had disclosed their HIV status to their partners. The average depression level was 9.11 ± 8.79 with about 23% meeting the threshold of positive depressive symptoms. The average total interpersonal support was 18.67 ± 6.61, with approximately 47.6% having weak interpersonal support.

### Adherence and associated factors

ART adherence was 34%. The distribution of missing data was not associated to adherence for the majority of independent variables (Table 2). Place of residence was associated with adherence in the univariate analysis, with 66.7% adherence of people residing in rural areas and 33.3% adherence among residents of urban areas (p = 0.0017) (Table 3). HIV status non-disclosure to partner was also associated with adherence, with 42% adherence among non-disclosed and 23% adherence among HIV-disclosed partners (p = 0.019). Availability of high interpersonal support was not statistically associated with adherence in the univariate analysis (p = 0.15). There was no association between adherence status and the following variables: relationship, gender, age, level of education, monthly income, and depression score. We note, however, that income was more likely to be reported (i.e., non-missing) by those who were adherent (p = 0.0013) (Table 2).

**Table 2:** Distribution of missing data of independent variables according to adherence to antiretroviral therapy in people living with HIV/AIDS (n = 159)

**Variables with missing data**	Adherence				p-value^a^
	No (n = 105)		Yes (n = 54)		
	**n**	%	**n**	%	
Relationship Status	0	0.0	1	1.8	0.34
Gender	30	28.6	10	18.5	0.18
Level of formal education	0	0.0	1	1.8	0.34
Monthly Income	4	3.8	11	20.4	0.0013*
HIV disclosure to partner	1	0.9	3	5.6	0.11

^a^ Two-tailed Fisher’s exact test;

**Table 3:** Univariable analysis of factors possibly associated with adherence to antiretroviral therapy in people living with HIV/AIDS (n = 159)

Variables	Adherence		Total	OR	95% CI		p-value
	n	%	n				
Gender							0.86^a^
Female	8	33.3	24	1			
Male	36	37.9	95	1.22	0.47	3.14	
Age (years)							0.63^b^
<30	5	38.5	13	1			
30–39	11	32.3	34	0.77	0.20	2.89	
40–49	13	27.1	48	0.59	0.16	2.15	
50–59	15	35.7	42	0.89	0.25	3.21	
60+	10	45.4	22	1.33	0.33	5.39	
Relationship Status							0.054^a,^*
Single/Widowed	30	42.3	71	1			
In Relationship	23	26.4	87	0.49	0.25	0.95	
Residence							0.0017^a,^**
Rural	36	66.7	77	1			
Urban	18	33.3	82	0.32	0.16	0.63	
Level of formal education							0.50^a^
None	7	13.0	17	1			
Primary	22	40.7	76	0.58	0.20	1.72	
Secondary	15	27.8	45	0.71	0.23	2.25	
Post-Secondary	9	16.7	20	1.17	0.32	4.32	
Monthly Income							1.00^a^
≤1000GH (ref)	37	30.1	123	1			
>1000GH	6	28.6	21	0.92	0.33	2.58	
HIV disclosure to partner							0.019^a,^*
Disclosed	17	23.0	74	1			
Have not disclosed	34	42.0	81	2.42	1.21	4.88	
Total Depression Score							
Not-depressed (< 16)	42	34.1	123	1			> 0.99^a^
Depressed (≥ 16)	12	33.3	36	0.96	0.44	2.12	
Interpersonal support (ISEL-12)							
Low (< 17)	21	27.6	76	1			0.15
High (≥ 17)	33	39.8	83	1.73	0.89	3.37	

^a^ Two-tailed Chi-squared test * p < 0.05; ** p < 0.0056

### Multivariable logistic regression estimates of adherence

In a stepwise multivariable logistic regression model with depression, residence, disclosure, and social support forced in the model (Table 4), we found that depression showed no association (p = 0.25), but those with high social support were more likely to be adherent (p = 0.033; aOR = 3.45, 95% CI = 1.09–5.88). The other factor associated with increased odds of adherence in the multivariable model was non-disclosure of HIV status (p = 0.044, aOR = 2.17, 95% CI = 1.03–4.54) and living in an urban area was also associated with decreased odds of adherence (p = 0.00037, aOR = 0.24, 95% CI = 0.11–0.52).

**Table 4:** Multivariable logistic regression of factors possibly associated with adherence to antiretroviral therapy in people living with HIV/AIDS (n = 159)

Variables	aOR	95% CI		p-value
		Lower	Upper	
Residence				
Rural (ref)	1	.	.	.
Urban	0.24	0.11	0.52	0.00037**
HIV disclosure to partner				
Disclosed (Ref)	1	-	-	-
Have not disclosed	2.17	1.03	4.54	0.044*
Depression status				
Not depressed	1	-	-	-
Depressed	0.56	0.22	1.47	0.25
Level of Social Support				
Low Social Support	1	-	-	-
High Social Support	3.45	1.09	5.88	0.033

aOR = adjusted odds ratio, ** p < 0.0056, * p < 0.05

Final model built using stepwise logistic regression (AIC) starting with depression and level of social support forced in (variables of primary interest) using variables that had a univariate association with adherence

## Discussion

In this study, we sought to investigate the association of depression and interpersonal support with adherence to ART among PLWH in the Volta region of Ghana. We found that a low percentage of respondents, 34%, were adherent to ART. This low rate of adherence falls short of the WHO 90-90-90 goals. Our findings indicate having high interpersonal support is associated with higher adherence, whereas disclosing HIV-status and living in urban areas were associated with a decreased odds of adherence to ART among PLWH.

The role of interpersonal support in ART adherence cannot be underestimated in sub-Saharan Africa [41]. Similar to our study, a study in Northern Ghana reported the benefits of interpersonal support in promoting adherence to ART among PLWH [42]. Our study further highlights the importance of interpersonal support by specifically observing the impact of interpersonal support on ART adherence. Interpersonal support includes the ability to find someone to help with daily chores when sick, provide physical support when stranded or when moving to a new apartment and having someone to take care of your house when away. Thus, the availability of interpersonal support are likely indicators that PLWH have someone that could physically be present to assist and remind them to take their ART, accompany them to follow-up visits, and/or serve as a surrogate to go for ART drug refills from health facilities. There was a minimal difference in the overall diagnostic disclosure rate ( i.e., 74 PLWH disclosed and 81 did not disclose their serological status) However, we found that the majority of the participants, who were adherent to ART, did not disclose their HIV status to their partners (23% disclosed and 42% did not disclose). Our bivariate results show a significant association between ART adherence and non-disclosure to a partner (aOR = 2.17; p = 0.04). This may mean that the PLWH are not able to receive the needed support from their spouse. Therefore, it is plausible interpersonal support helps sustain treatment linkage and promotes sustained adherence to ART that otherwise would have been interrupted. Previous studies [43, 44] have also found that PLWH who disclosed their HIV status to their partners are more likely to adhere to their ART and achieve better treatment outcomes. Given these documented benefits, an intervention to strengthen interpersonal support could help improve ART adherence outcomes among PLHW, especially those in urban areas, concurrent with findings of previous interpersonal support interventions [25, 45]. However, this finding is contrary to the results of a recent study in the Greater Accra Region of Ghana and Uganda, which showed interpersonal support from family and friends had no association with adherence [21, 46].

Depression is known to negatively affect adherence to ART [47], but we found no significant association in the present study. This discrepancy may be attributable to the small sample size used in the present study resulting in the inability to detect true differences. Another plausible factor is the cultural validity of the CES-D tool (20-item) in the Ghanaian context. Although the CES-D scale has been used in other studies in Ghana [48, 49], it has not been culturally validated. A mixed-method study of its related scale (CES-D 10), for example, revealed limitations with cultural validity, despite demonstrating adequate reliability and validity on quantitative analysis [50]. In addition, our findings found no association of post-secondary education status with ART adherence, unlike other studies in SSA [51, 52], which might also reflect the sample size.

Urban residence was found to be associated with decreased odds of adherence. This observation is comparable to findings of a systematic review in SSA [41]. Unlike rural residents who have more community support in Ghana, urban lifestyle is often individualistic with minimal communal support [53]. Therefore, urban PLWH may lack the support network needed to remind and support them to adhere to ART—as underscored by our findings, high social support is associated with adherence. This coupled with the general stress and high cost of living for urban residents may impact their adherence to ART. Interestingly, observations made in Kenya by Mukui and colleagues [54], reported lower rates of adherence for rural residence. This might however be due to differences in rural versus urban settlements in Ghana and Kenya.

We found that people adherence to ART were more likely to not disclose their HIV status to their partners. In a study conducted in Uganda [55], PLWH have reported how they experience verbal and physical abuse from their husband, co-wife and sometimes mothers because of their HIV positive status. Some participants reported that their partners even prevented them from taking their medications [55]. Our findings suggest a need for intervention to reduce stigma and discrimination toward PLWH among the public especially those whose partners are living with HIV.

Our study had important limitations. We used purposive sampling to recruit participants into our study hence the results of our study cannot be generalized. Additionally, our sample size (*n* = 159) was relatively small, thus affecting our power to detect all predictors of adherence. Like all self-report data, some responses might be biased toward social desirability, particularly when a survey is administered face-to-face. Moreover, because we recruited participants from a single clinic in the Volta region of Ghana, we are unable to generalize our findings to those outside this region, nonetheless, the findings are compelling enough to inform similar studies on a national scale. We recommend that future studies use larger randomly selected patient samples to improve internal and external validity. Moreover, our study used a cross-sectional design and we do not know the temporal sequence between our outcome and exposures. Therefore, any observed significant relationship between key variables do not imply causality. Another limitation was the challenge with large amount of missing data, which has a potential to bias our results if missing not at random (MNAR), e.g., if those with the lowest income are more likely not to report income. The high amount of missing data might be due to some research assistants omitting some variables during data collection. Nevertheless, we ran a multiple imputation approach to utilize all participants with adherence outcome data, which is valid under a missing at random (MAR) assumption.

## Conclusion

Our study demonstrated that having high interpersonal social support, not disclosure status to partner and living in rural area were independent predictors of adherence of ART among PLWH. The findings highlight the need to develop and implement more novel and robust interpersonal support services for PLWH. Moreover, there is a need for assessment of the availability of tangible interpersonal support services during clinical encounters and the need for follow-up on patients with no tangible interpersonal support to improve ART adherence among PLWH. Future studies can explore the national, regional, and sub-regional variations in social support and how this impact ART adherence among PLWH.

## Data Availability

All data supporting the results and conclusion of this paper are included in the article.

## List of abbreviations

AIDS: Acquired Immune Deficiency Syndrome
ART: Antiretroviral Treatment
CES-D: Center for Epidemiologic Studies Depression Scale
CI: Confidence Interval
HIV: Human Immunodeficiency Virus
ISEL: Interpersonal Support Evaluation List
OR: Odds Ratio
PLWH: People Living with HIV
SSA: Sub-Sharan Africa
STI: Sexually Transmitted Infection
WHO: World Health Organization
